# Ultrasonography validation for early alteration of diaphragm echodensity and function in the *mdx* mouse model of Duchenne muscular dystrophy

**DOI:** 10.1371/journal.pone.0245397

**Published:** 2021-01-12

**Authors:** Antonietta Mele, Paola Mantuano, Adriano Fonzino, Francesco Rana, Roberta Francesca Capogrosso, Francesca Sanarica, Jean-Francois Rolland, Ornella Cappellari, Annamaria De Luca

**Affiliations:** 1 Section of Pharmacology, Department of Pharmacy—Drug Sciences, University of Bari "Aldo Moro", Bari, Italy; 2 Axxam, S.p.A, Bresso, Milan, Italy; University of Tennessee Health Science Center College of Graduate Health Sciences, UNITED STATES

## Abstract

The *mdx* mouse model of Duchenne muscular dystrophy is characterized by functional and structural alterations of the diaphragm since early stages of pathology, closely resembling patients’ condition. In recent years, ultrasonography has been proposed as a useful longitudinal non-invasive technique to assess *mdx* diaphragm dysfunction and evaluate drug efficacy over time. To date, only a few preclinical studies have been conducted. Therefore, an independent validation of this method by different laboratories is needed to increase results reliability and reduce biases. Here, we performed diaphragm ultrasonography in 3- and 6-month-old *mdx* mice, the preferred age-window for pharmacology studies. The alteration of diaphragm function over time was measured as diaphragm ultrasound movement amplitude. At the same time points, a first-time assessment of diaphragm echodensity was performed, as an experimental index of progressive loss of contractile tissue. A parallel evaluation of other *in vivo* and *ex vivo* dystrophy-relevant readouts was carried out. Both 3- and 6-month-old *mdx* mice showed a significant decrease in diaphragm amplitude compared to wild type (wt) mice. This index was well-correlated either with *in vivo* running performance or *ex vivo* isometric tetanic force of isolated diaphragm. In addition, diaphragms from 6-month-old dystrophic mice were also highly susceptible to eccentric contraction *ex vivo*. Importantly, we disclosed an age-dependent increase in echodensity in *mdx* mice not observed in wt animals, which was independent from abdominal wall thickness. This was accompanied by a notable increase of pro-fibrotic TGF-β1 levels in the *mdx* diaphragm and of non-muscle tissue amount in diaphragm sections stained by hematoxylin & eosin. Our findings corroborate the usefulness of diaphragm ultrasonography in preclinical drug studies as a powerful tool to monitor *mdx* pathology progression since early stages.

## Introduction

The dystrophic *mdx* mouse is a widely used animal model for Duchenne muscular dystrophy (DMD) in preclinical studies, which shares the same biochemical defect of DMD patients for an X-linked stop codon mutation in the *DMD* gene encoding for dystrophin protein [1–3]. The *mdx* mouse develops an early degenerative phase in hind limb muscles around the 3^rd^– 4^th^ week of age, which is efficiently overcome by an effective regeneration process. Around the 12^th^– 16^th^ week, *mdx* muscle pathology stabilizes to a state of low chronic damage [1–3]. Most pharmacological studies are conducted in this age window, in order to mimic the stage at which pharmacological interventions become necessary in DMD boys. Consequently, the scientific community made a great effort to identify the best standard operating procedures and the best primary readout parameters to assess drug/treatment effectiveness [1–4]. Longitudinal *in vivo* readouts of muscle performance allows to monitor pathology progression over time and to assess beneficial effects, as well as unwanted side effects, of potential therapeutics. However, *in vivo* measurements commonly used in studies with *mdx* mice, such as the forelimb grip strength test as a measure of weakness and performance to exercise to assess fatigue, cannot be considered as parameters purely related to muscular function, since they are influenced by other stimuli (*i*.*e*. peripheral nervous system, vascular reactivity) and animal willingness [1–4]. In 2016, Whitehead and colleagues described the possibility to monitor diaphragm function *in vivo* in *mdx* mice by means of ultrasonography, a non-invasive technique that had been mainly used to detect late changes in cardiac function in this murine model [5]. Such an ultrasonographic alteration of diaphragm function was well-correlated with the impairment of diaphragm force measured *ex vivo*, supporting the value of this approach for translational studies [5]. In fact, it is well-known that *mdx* diaphragm shows a progression of dystrophic pathology which closely resembles the one observed in humans, and its functional and structural alteration contributes to respiratory impairment in dystrophic subjects [1, 6]. Ultrasonography provides the advantage to offer non-invasive and longitudinal endpoints in dystrophic animal models, which are easily translatable to the clinical field. In fact, a morphological and functional ultrasound evaluation of skeletal muscles, including diaphragm, has been promisingly conducted in several clinical studies on DMD patients [7–10].

In light of these observations, the present study was aimed to perform an independent validation of the ultrasound technique for the longitudinal detection of diaphragm alterations in *mdx* mice. The need of an independent validation of preclinical approaches by different laboratories adheres to commonly accepted guidelines, as this helps to verify reproducibility of data and to reduce biases, meanwhile opening towards novel useful readouts.

In this regard, our study was also aimed at assessing, for the first time, the possible age-related changes in diaphragm echodensity as an index of pathology progression and loss of contractile tissue. Echodensity measurements may represent an additional readout for the preclinical assessment of early efficacy of therapeutics in *mdx* mice aimed to reduce progressive fibrosis, an intense field of effort by the community. In addition, the longitudinal assessment of echodensity may help to distinguish intrinsic primary diaphragm structural alterations from the adaptive dysfunction occurring as an early consequence of subclinical changes in left ventricular function, which progressively lead to late cardiac failure in *mdx* mice [11, 12], providing a further mean to prioritize preclinical research of therapeutics. We focused on mice of 3 and 6 months of age, representing the time window where most pharmacological studies are conducted. *In vivo* and *ex vivo* readouts were assessed in parallel, in order to obtain a better correlation with other major pathology features.

## Materials and methods

### Experimental groups

All the experiments performed in this study were conducted in conformity with the Italian Guidelines for Care and Use of Laboratory Animals (D.L.116/92), and with the European Directive (2010/63/UE). The study has been approved by national ethic committee for research animal welfare of the Italian Ministry of Health (authorization numbers: 815 and 816/2017-PR). Experimental protocols conform to the standard operating procedures (SOPs) for preclinical tests in *mdx* mice, available on the TREAT–NMD website (*http*:*//www*.*treat-nmd*.*eu/research/preclinical/dmd-sops/*).

A total of 36 male mice, 18 wild type (wt; C57BL/10ScSn/J) and 18 *mdx* (C57BL/10ScSn-*Dmd*^mdx^/J) of 8 weeks of age were purchased from The Jackson Laboratory (USA, distributed by Charles River, Calco, Italy) to perform the study. For each genotype, the animals were housed into cages based on mean body weight and then randomly assigned to different experimental groups.

All mice were acclimatized for one week in the animal facility, prior to the initiation of the experimental protocol. For the whole duration of the study, animals were maintained on a controlled diet, with a daily amount of chow of 4–5 g/mouse (for composition, see Capogrosso *et al*., 2018 [13]). No signs of stress (lack of appetite, body weight loss, hair loss, stereotypic or aggressive behavior, *etc*.) or macroscopic alterations of vital functions were observed in mice of either genotype throughout the experimental phase. Longitudinal, non-invasive *in vivo* experiments (diaphragm ultrasonography and echocardiography, forelimb grip strength test, exhaustion test on a horizontal treadmill) were performed on wt (n = 9) and *mdx* (n = 9) mice, both at the start (3 months of age; M3) and at the end (6 months of age; M6) of the study. At M6, these mice were sacrificed for terminal *ex vivo* diaphragm force measurements. In parallel, two cohorts of wt (n = 9) and *mdx* (n = 9) mice were used to perform *ex vivo* assessments at M3.

### Diaphragm ultrasonography and echocardiography

At the beginning (M3) and at the end (M6) of the experimental protocol, ultrasound imaging of the diaphragm and echocardiography were performed on wt and *mdx* mice by using the ultra-high frequency ultrasound biomicroscopy system Vevo^®^ 2100 (VisualSonics, Toronto, ON, Canada) [14, 15]. The animals were properly prepared prior to each imaging session to allow optimal image acquisition. Each mouse was anaesthetized via inhalation (~ 3% isoflurane and 1.5% O_2_ l/min during the induction phase, then constantly maintained via nose cone at ~ 2% isoflurane and 1.5% O_2_) and placed on a thermostatically controlled table (kept at 37°C). This platform was equipped with four copper leads to allow the monitoring of both heart and respiratory rate of the mouse, which minimizes any physiological variation. Body temperature was also constantly monitored during the imaging session using a rectal probe. A petroleum-based lubricant was placed on each eye in order to prevent drying of the area. The chest and the abdomen of the mouse were shaved with depilatory cream in order to avoid any interference during the acquisition of the images. A small amount of pre-heated ultrasound gel was applied between the animal skin and the probe to guarantee proper image recording, as well as on the platform copper leads. During each imaging session, a minimal pressure was applied to minimize distortions.

**Fig 1:**
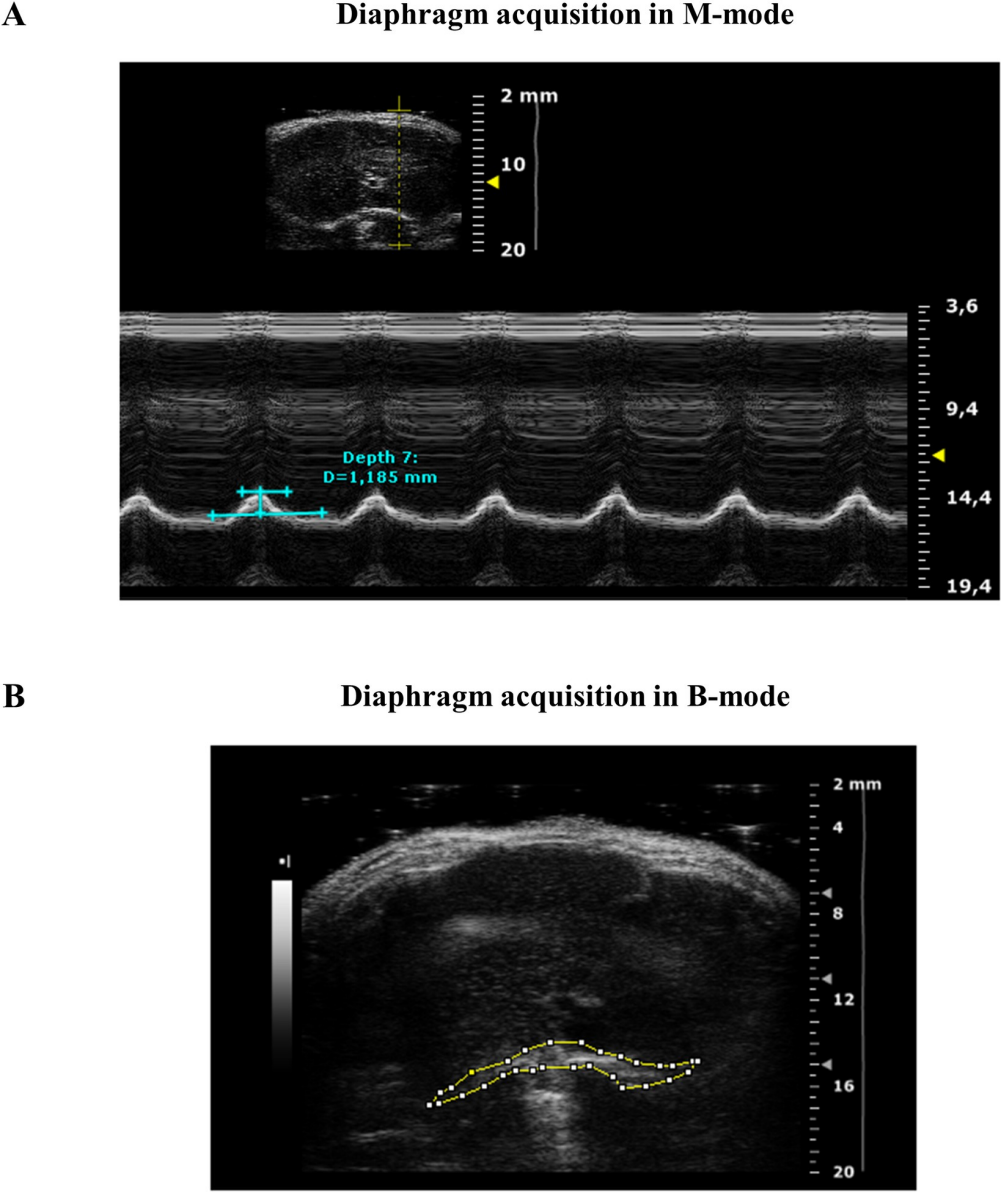
Sample images of ultrasonography diaphragm acquisition. The figure shows representative frames of the diaphragm acquired in M-mode (**A**) and B-mode (**B**), by ultrasonography. Diaphragm amplitude was measured in M-mode as the distance in mm, directly provided by the Vevo2100 software, between the baseline and the peak of contraction. The B-mode acquisition was used to perform echodensity analysis performed by using the Image J software. Mean pixel echodensity was obtained from the analysis of the outlined diaphragm section of constant dimensions of 4514.0 ± 17.6 pixels.

For diaphragm ultrasonography, as described by Whitehead *et al*. [5], each mouse was placed in dorsal decubitus position and the platform was tilted by 30° from horizontal with the head of the mouse lower with respect to the feet. The probe was fixed transversally to the mid-sternal of the mouse at 120° with respect to the platform. Image acquisition was performed in mono-dimensional (M-Mode) and bi-dimensional (B-Mode) (**Fig 1A and 1B**) by using a probe operating at a frequency of 21 MHz characterized by a lateral and axial resolution of 165 and 75 μm, respectively. As indicated by Whitehead *et al*., diaphragm movement amplitude was measured in M-Mode during normal breathing cycles on the left side, which provides less variability in measurements for both wt and *mdx* mice. The amplitude during each inspiration (positive deflection) was measured as the distance (in mm) between the baseline and the peak of contraction (**Fig 1A**). For each mouse, diaphragm amplitude was calculated as the mean value obtained from 3–5 measurements. Images acquired in B-Mode (**Fig 1B**) were used to evaluate diaphragm echodensity, which was measured using ImageJ^®^ software by creating a grey scale analysis histogram on the entire outlined diaphragm section of a constant dimensions of 4514.0 ± 17.6 pixels. In particular, for each mouse, diaphragm echodensity was obtained as the main value obtained from 4 frames of the same acquisition drawing the ROI in the same area of the diaphragm. Echodensity differences were expressed as the percentage difference between wt and *mdx* groups of the mean echo intensity of the pixels included in the outlined area (**Fig 1B**).

Echocardiography was performed on wt and *mdx* mice in M-Mode, by using a high–resolution transducer at a frequency of 40 MHz. Images were acquired in modified parasternal long axis view (PLAX) with the animal placed in supine position. Each parameter was acquired and calculated according to TREAT–NMD SOPs. Briefly, after M-mode image acquisitions, the left ventricular (LV) M-Mode trace was used to measure functional parameters such as stroke volume (SV, μl), cardiac output (CO, ml/min), ejection fraction (EF, %), fractional shortening (FS, %), and structural parameters such as diastolic interventricular septal wall thickness (IVS;d, mm), systolic interventricular septal wall thickness (IVS;s, mm), LV end-diastolic diameter (LVID;d, mm), LV end-systolic diameter (LVID;s, mm), LV diastolic posterior wall thickness (LVPW;d, mm), LV systolic posterior wall thickness (LVPW;s, mm) and LV mass (mg). [16, 17].

To avoid intra- and inter-variability, ultrasound data analysis was performed by two different operators twice. The data sets obtained from repeated analyses were consistently overlapping. The reproducibility and reliability of these analyses was verified by performing a Fisher’s F-test for the analysis of variance, followed by an independent Student’s t-test; these tests allowed us to exclude any statistically significant difference between data sets of individual operators.

### Abdominal wall thickness assessment to verify ultrasound attenuation

**Fig 2:**
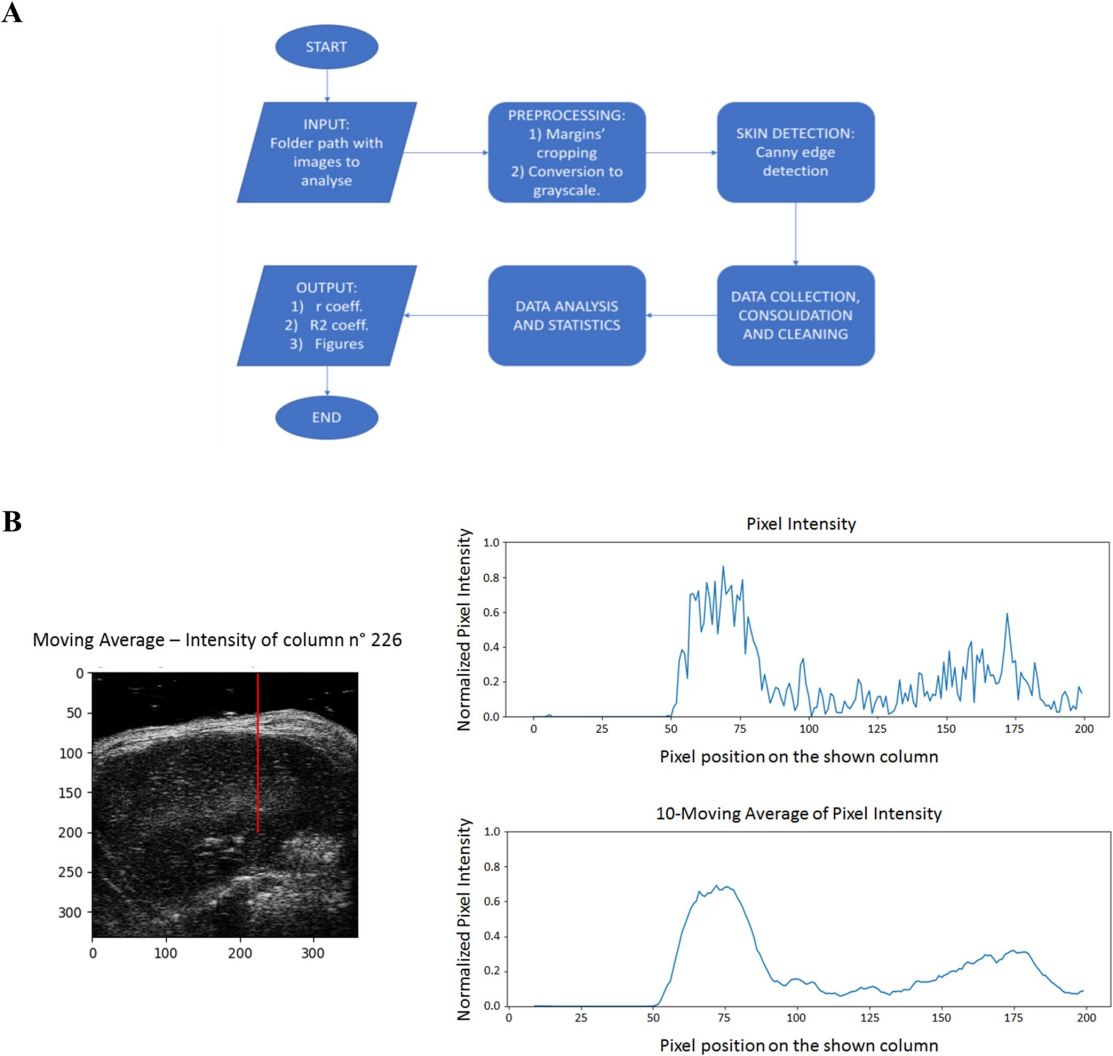
Analysis of hyperechoic tissue. In **A**, is shown a schematic representation of the analysis performed to evaluate the possible contribution of hyperechoic upper layer of tissue (abdominal wall) on signal’s attenuation of deeper layers. In **B**, is shown a sample image of the algorithm that scans the upper portion of the images, one column at a time, retrieving information from these pixels. The image on the left shows an ultrasonography acquisition where the red cursor indicates the column of pixel analyzed at that moment. The line charts on the right show Normalized Pixel Intensity sampled by the red cursor (up) and 10-Moving Average of Pixel Intensity of pixels sampled by the red cursor (down).

To better validate echodensity measurements, we developed a method to establish the role of the hyperechoic superficial abdominal wall (AW) thickness in ultrasonography signal penetration [18, 19]. In particular, a computational analysis was performed on 36 B-Mode images randomly selected from those used for echodensity measurements. The main steps of the analysis are shown in **Fig 2A**. First, images were preprocessed cropping the useless borders, converting them from Red-green-blue-alpha (RGBA) to grayscale (pixel intensity from 0 to 255), and then normalizing it to maximal values taken as 1. After that, normalized cropped grayscale images where analyzed by the Canny Edge Detection Algorithm after a preliminary application of a Gaussian Blur Filter [20, 21]. Results of Canny step allowed to define both edges of hyper echogenic layers due to abdominal wall for each column of pixels taken sequentially for the analysis, and then to calculate *AW Thickness (number of pixels; mean value +/-)*, *AW Mean Pixel Intensity* (P.I.), and *Below-AW P*.*I*. (mean P.I. of the underlying 50 pixels) (**Fig 2B**). The univocal detection of edges with *AW thickness* above 5 and below 50 pixels was a priority criteria. Data obtained for every image were consolidated into a single big data frame. Then, we computed the linear correlation coefficient (*Pearson Correlation Coefficient*, r) and the *Determination Coefficient* (R^2^) between the ratio of *Below-AW P*.*I*. */ AW P*.*I*. and the *AW Thickness*. The analysis was performed by the *Python* program language *version 3*.*7*.*4* [22], using libraries like *Scipy*, *Sklearn*, *Skimage*, *Matplotlib*.*Pyplot* and *Seaborn* from the scientific package Anaco*nda distribution version 2019*.*10* (https://docs.anaconda.com/anaconda-cloud/faq/#what-is-anaconda-inc).

The code is available in S1 File as HTML file of the Jupyter Notebook.

### Forelimb grip strength test and exhaustion test

At the same time points of ultrasonography evaluations (M3 and M6), all animals were monitored for body weight and forelimb strength by means of a grip strength meter (Columbus Instruments, Columbus, Ohio, USA). The values of both absolute and normalized (to body weight) maximal forelimb force were used for statistical analysis [13, 17, 23–26]. In parallel, an acute exercise resistance test on a horizontal treadmill (Columbus Instruments) was performed, as an *in vivo* index of animal fatigability. All mice were made running for 5 mins at 5 m/min, then increasing the speed of 1 m/min each minute. The total distance (in meters) run by each mouse until exhaustion was calculated [13, 17, 23–26].

### *Ex vivo* isometric and eccentric contraction recordings of diaphragm

At M6, after the completion of *in vivo* experiments all mice were sacrificed to perform *ex vivo* measurements on diaphragm muscle. In particular, isometric and eccentric contraction recordings on isolated diaphragm strips were performed. In parallel, a cohort of wt and one of *mdx* mice were sacrificed to perform the same protocol at 3 months of age (M3). The diaphragm was excised from each mouse deeply anaesthetized with an intraperitoneal injection of a mixture of ketamine (100 mg/kg) and xylazine (16 mg/kg). A strip of right hemi-diaphragm (no more than 4 mm wide) was cut from the excised muscle, firmly tied at the rib and at the central tendon, and placed into a vertical muscle bath containing a normal physiological Ringer’s solution (in mM): NaCl 148, KCl 4.5, CaCl_2_ 2.0/2.5, MgCl_2_ 1.0, NaH_2_PO_4_ 0.44, NaHCO_3_ 12.0, glucose 5.55, pH 7.2–7.4, 27 ± 1°C) continuously gassed with a mixture of 95% O_2_ and 5% CO_2_. The central tendon of the diaphragm strip was fixed to a chamber hook, while the rib was fixed to a Dual-Mode Lever System force transducer (300C-LR, Aurora Scientific Inc., ON, Canada) connected to a proper interface and data acquisition system (604A, ASI with Dynamic Muscle Control software DMCv4.1.6, Aurora Scientific Inc., ON, Canada). Electrical field stimulation was obtained by two axial platinum electrodes which closely flanked the diaphragm, connected to a stimulator (LE 12406, 2Biological Instruments, VA, Italy). After equilibration (~ 30 min), the diaphragm preparation was stretched to its optimal length (L_0_, measured with an external caliper), which is the length producing the maximal single contraction (twitch, Ptw) in response to a 0.2 ms square wave 40–60 mV pulse. Maximal twitch tension was obtained as the mean value from 5 twitches elicited by pulses of 0.2 ms, every 30 seconds. Tetanic contractions were elicited by applying trains of 450 ms of 2.0 ms pulses at increasing frequencies (10–200 Hz), every 2 minutes. Maximal tetanic force (P0) was usually recorded at 140–180 Hz. At the end of isometric recordings, the diaphragm was subjected to a series of 10 eccentric contractions at 120 Hz for 500 ms, every 30 seconds. An initial isometric contraction was elicited for 300 ms, followed by a stretch of 10% L_0_ at a speed of 1L_0_ s^−1^ imposed for the last 200 ms. The progressive decay in isometric force at 5^th^ and 10^th^ pulse was calculated as percentage of reduction compared to force produced at the 1^st^ pulse. Two tetanic stimuli (120 Hz, 500ms), were elicited 4 and 30 minutes after the eccentric contraction protocol, to calculate the recovery from the stretch-induced force drop compared to the tetanic force registered before the protocol started, as well as muscle compliance to stretch. Data were analyzed using the Dynamic Muscle Analysis software v3.2 (ASI) to obtain Ptw and P0 values, then normalized to muscle cross sectional area according to the equation sP = P/(Mass/Lf*D) where P is absolute tension, Mass is the muscle mass, D is the density of skeletal muscle (1.06 g/cm^3^), Lf was determined by multiplying L_0_ by previously determined muscle length to fiber length ratio (for diaphragm = 1) [13, 17, 23, 24].

### Determination of TGF-β1 levels in the diaphragm

Another portion of the right hemi-diaphragm was excised from 3-month-old and 6-month-old wt and *mdx* mice, immediately snap frozen in liquid nitrogen and stored at -80°C until being used to measure pro-fibrotic TGF-β1 (Transforming Growth Factor—β1) levels by enzyme linked immunosorbent assay (ELISA). The remaining diaphragm tissue was mounted and frozen for histological analysis. Briefly, diaphragm portions of 15–30 μg each, were homogenized in micro centrifuge tubes containing 200–300 μl of freshly-prepared lysis buffer (20 mM TRIS pH 8.0, 1% Triton X-100, 137 mM sodium chloride, 10% glycerol, 5 mM ethylenediaminetetraacetic acid and 1 mM phenyl methyl sulphonyl fluoride). Tissue homogenates were then centrifuged (10 min, 12000 rpm, 4°C) and supernatants were used for the ELISA (Quantikine® ELISA Mouse TGF-β1 Immunoassay, R&D Biosystems) according to manufacturer’s instructions. TGF-β1 levels were expressed as pg of TGF-β1/μg of total protein content [23, 24].

### Histological analysis

At sacrifice, diaphragm strips from wt and *mdx* mice were rolled and mounted on cork with a small amount of Tissue–Tek O.C.T. (Bio–Optica, Milan, Italy) and then immersed in isopentane cooled with liquid nitrogen (N_2_) for 60 seconds and stored at -80°C until further processing for histology. Serial cross-sections (6–8 μm thick) from each frozen diaphragm were transversally cut into a cryostat microtome set at -20°C (HM 525 NX, Thermo Fisher Scientific, MA, USA) on proper glass slides (Superfrost™ Plus, Thermo Fisher Scientific). The prepared sections underwent the classical hematoxylin & eosin (H&E) staining and Masson's trichrome staining to evaluate skeletal muscle architecture according to TREAT-NMD SOPs, as described in detail in previous work [13, 17, 23–25, 27].

### Statistical analysis

All experimental data are expressed as the mean ± the standard error of the mean (SEM). Multiple statistical comparison among groups was performed by one-way analysis of variance (ANOVA), followed by Bonferroni post hoc correction to evaluate intra- and inter-group variability and to avoid false positives. A direct comparison between data pairs was made by unpaired Student’s t-test, when necessary.

## Results

### Ultrasonography evaluation of diaphragm and heart

**Fig 3:**
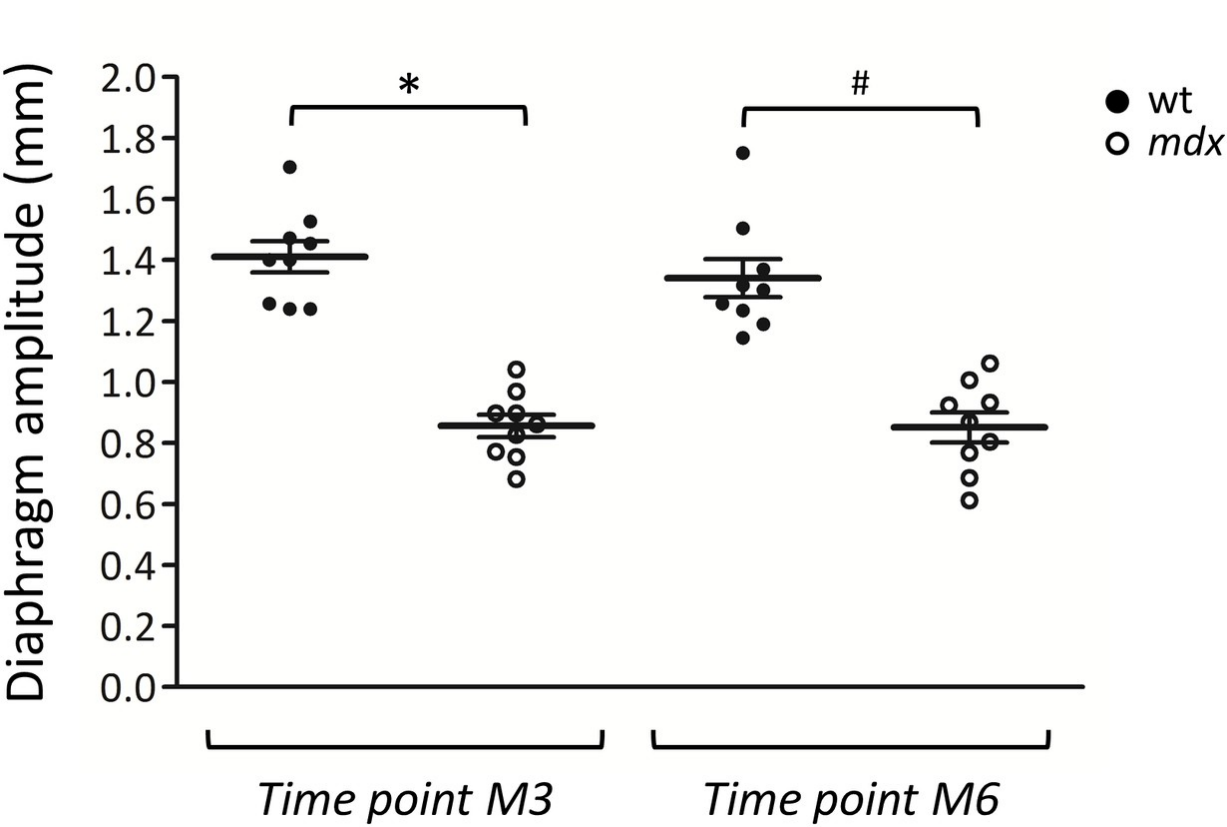
Measurement of diaphragm amplitude by ultrasonography. The scatter plot graph illustrates individual mouse values, mean and standard error of the mean (SEM) obtained for diaphragm amplitude in wt (●) and *mdx* (○) mice at 3 (M3) and 6 (M6) months of age. A statistically significant difference among groups was found by ANOVA (F = 35.7, p < 2.5 x 10^−10^). Bonferroni post hoc correction for individual differences between groups is as follows: significantly different vs * wt M3 (p < 7.9 x 10^−9^), ^#^ wt M6 (p < 9.9 x 10^−8^).

Diaphragm movement amplitude was measured by ultrasonography in wt and *mdx* mice at M3 and M6 (**Fig 3**). At M3, a significant reduction in diaphragm amplitude (– 39.3%) was found in *mdx* mice compared to age-matched wt mice. Similarly, at M6, *mdx* mice exhibited a significantly reduced diaphragm amplitude equal to– 36.5% with respect to wt counterpart. No significant age-related change of this parameter was observed in wt mice between M3 and M6 (**Fig 3**).

**Fig 4:**
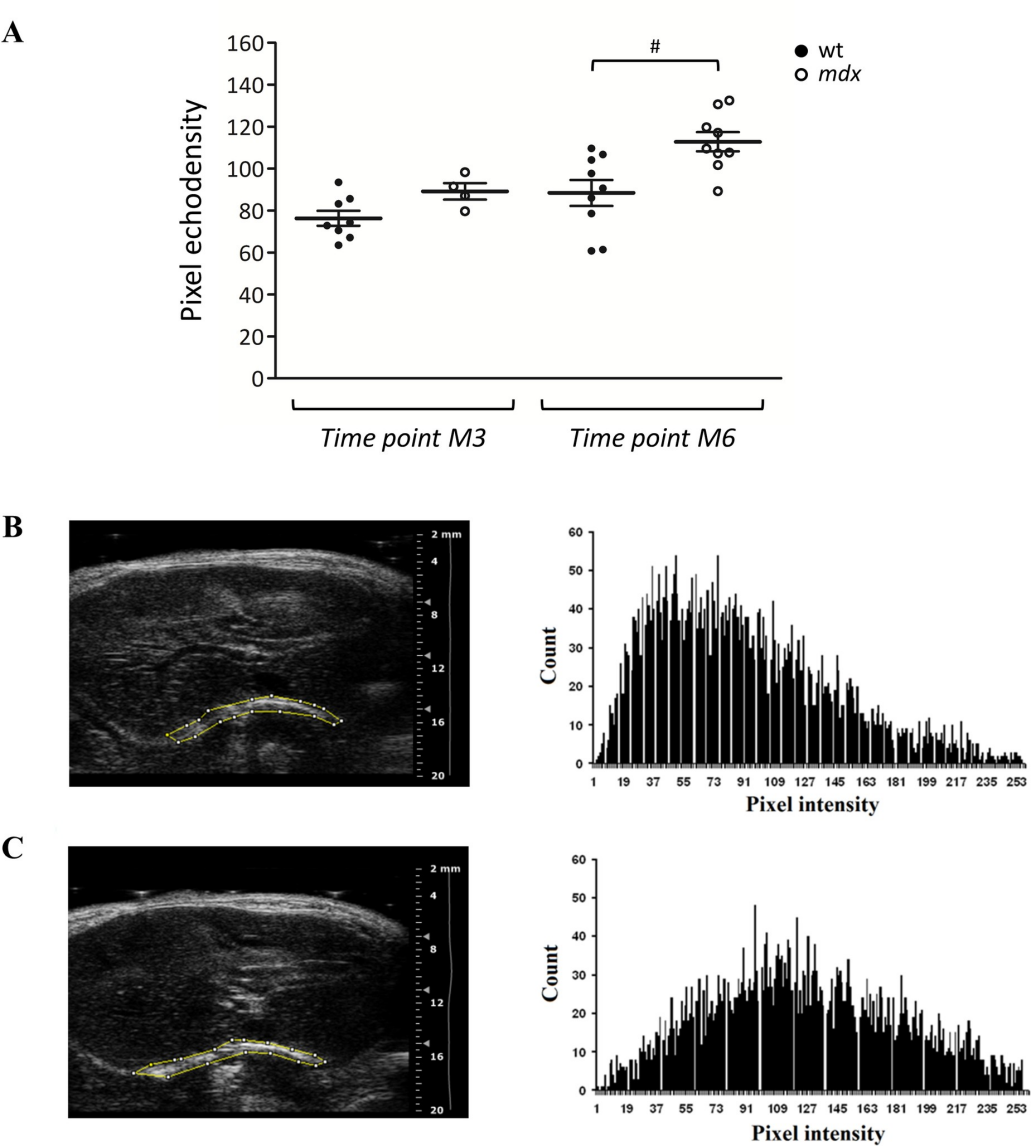
Diaphragm echodensity evaluation. The scatter plot graph in **A** shows individual mouse values, mean and standard error of the mean (SEM) obtained for pixel echodensity in wt (●) and *mdx* (○) mice at 3 (M3) and 6 (M6) months of age. A statistically significant difference among groups was found by ANOVA at M6 (F = 5.08, p < 0.007). Bonferroni post hoc correction for individual differences between groups is as follows: significantly different vs ^#^ wt M6 (p < 0.003). In **B** and **C** are shown the histograms of pixel distribution (right panel) obtained by measuring echodensity in wt (**B**) and *mdx* (**C**) mice at M6, with sample images used for analysis (left panel). Each histogram represents the number of repetitions (count; reported on the Y axis) of each pixel value (pixel intensity; indicated on the X axis).

At the same time points (M3, M6), diaphragm echodensity was also evaluated for both genotypes (**Fig 4A–4C**). No significant change in mean pixel echodensity was observed in a preliminary assessment at M3 between wt and *mdx* mice. However, a trend toward increase (+ 19.7%) was observed in *mdx* mice, which pushed us to perform a more detailed analysis at 6 months of age. Interestingly, at M6, the mean echo intensity of pixels included in the outlined area was of 88.40 ± 6.15 (n = 9) in wt and 112.82 ± 4.59 (n = 9) in *mdx* mice, resulting in a significant increase of this parameter of + 27.6% (**Fig 4A**). The gray scale histogram obtained via *ImageJ* software showed a clearly different distribution of pixel intensity at M6 between wt and *mdx* mice (**Fig 4B and 4C**).

**Fig 5:**
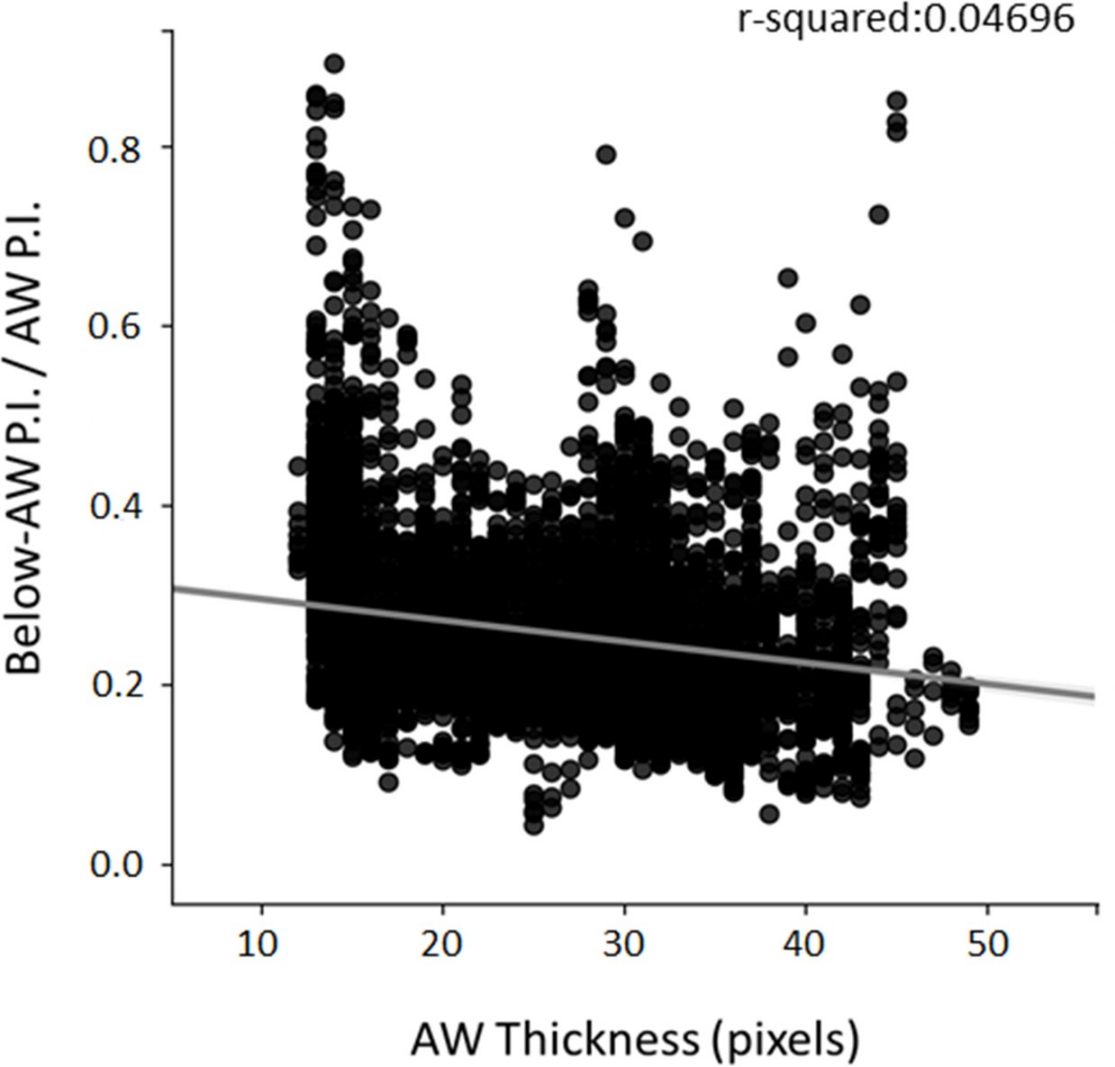
Evaluation of abdominal wall thickness during ultrasound measurements. Correlation between AW (abdominal wall) Thickness detected with Canny Edge Detection and the Below-AW P.I. (pixel intensity) / AW P.I. ratio (mean). On the top, on the right of the chart, is showed the R^2^ (r-squared).

The possible ultrasound attenuation due to abdominal wall (AW) was also assessed. To this aim, a total of 6473 columns were analyzed from all the 36 images, for a total of 480833 pixels. Collected and consolidated data allowed to search for a possible correlation between *Below-AW P*.*I*. / *AW P*.*I*. and the *AW Thickness*. As shown in **Fig 5**, a weak negative linear correlation (r = - 0.2167) was found likely to be negligible, since it is able to explain only less than 5% of the variations of the data (R^2^ = 0.04696). Importantly, echocardiography performed at M6 showed that the main cardiac parameters related to left ventricular function, stroke volume (μl), cardiac output (ml/min), ejection fraction (%) and fractional shortening (%), were not significantly modified in *mdx* mice with respect to age-matched wt mice (**Table 1**). In parallel, no significant alterations were found for heart morphology, with no changes in diastolic interventricular septal wall thickness (IVS;d, mm), systolic interventricular septal wall thickness (IVS;s, mm), LV end-diastolic diameter (LVID;d, mm), LV end-systolic diameter (LVID;s, mm), LV diastolic posterior wall thickness (LVPW;d, mm), LV systolic posterior wall thickness (LVPW;s, mm) and LV mass (mg).

**Table 1:** Echocardiography.

LV Parameters (M6)	wt (n = 9)	*mdx* (n = 9)
SV (μl)	34.3 ± *1*.*0*	35.0 ± *1*.*8*
CO (ml/min)	15.7 ± *0*.*6*	14.0 ± *1*.*1*
EF (%)	62.6 ± *1*.*2*	61.5 ± *1*.*8*
FS (%)	33.2 ± *0*.*8*	32.5 ± *1*.*3*
IVS;d (mm)	0.96 ± *0*.*06*	0.97 ± *0*.*03*
IVS;s (mm)	1.35 ± *0*.*08*	1.37 ± *0*.*05*
LVID;d (mm)	3.63 ± *0*.*07*	3.72 ± *0*.*08*
LVID;s (mm)	2.50 ± *0*.*13*	2.57 ± *0*.*10*
LVPW;d (mm)	0.92 ± *0*.*03*	1.03 ± *0*.*04*
LVPW;s (mm)	1.23 ± *0*.*05*	1.33 ± *0*.*05*
LV mass (mg)	123.7 ± *7*.*4*	142.2 ± *7*.*3*

The table shows the parameters of left ventricular function and structure measured by echocardiography in *mdx* mice compared to wt mice at 6 months of age (M6). All values are expressed as mean ± SEM from the number of mice indicated in brackets. No statistically significant differences among groups were found by unpaired Student’s t-test (with p values > 0.1). Abbreviations: Stroke Volume (SV, μl), Cardiac Output (CO, ml/min), Ejection Fraction (EF, %), Fractional Shortening (FS, %), diastolic and systolic interventricular septal wall thickness (IVS;d and IVS;s, mm), left ventricular (LV) end-diastolic and end-systrolic diameter (LVID;d and LVID;s, mm), LV diastolic and systolic posterior wall thickness (LVPW;d and LVPW;s, mm), LV mass (mg).

### *In vivo* forelimb force and exercise performance

As shown in **Table 2**, a physiological, age-dependent increase in body weight was observed in both *mdx* and wt mice groups throughout the experimental protocol, with no significant differences between the two genotypes at each time point. In line with our previous studies [13, 17, 23–25], *mdx* mice had significantly lower values of forelimb grip strength, either absolute or normalized to body weight, in comparison to wt mice, both at M3 and M6 (**Table 2**). In *mdx* mice, force production at M6 was reduced of– 11.5% and of– 26.6% for absolute and normalized values, respectively, in comparison to M3, while the observed percentage reduction was modest in wt mice.

**Table 2:** *In vivo* force and exercise performance.

Time point	Group	BW (g)	Fmax (KGF)	Δ Fmax M6 vs M3	Fmax/BW (KGF/kg)	Δ Fmax/BW M6 vs M3	*e*-test (m)	Δ *e*-test M6 vs M3
**M3**	wt (n = 9)	28.2 ± *0*.*5*	0.186 ± *0*.*005*	-	6.6 ± *0*.*16*	-	430 ± *35*	-
	*mdx* (n = 9)	28 ± *0*.*4*	0.139 ± *0*.*004* *	-	4.9 ± *0*.*14* *	-	269 ± *19* *	-
**M6**	wt (n = 9)	33.5 ± *0*.*7*	0.182 ± *0*.*006*	- 2.2%	5.5 ± *0*.*21*	- 16.7%	491 ± *46*	+ 22.1%
	*mdx* (n = 9)	34.1 ± *0*.*4*	0.123 ± *0*.*003* ^#^	- 11.5%	3.6 ± *0*.*16* ^#^	- 26.6%	183 ± *16* ^#^	- 32%

The table shows the values of body weight (BW, g), maximal forelimb force either absolute (Fmax, KGF) or normalized to BW (Fmax/BW, KGF/kg), and total distance (m) run during an exhaustion test (*e*-test) on treadmill for 3-month-old (M3) and 6-month-old (M6) wt and *mdx* mice. The percentage variation (Δ, %), for Fmax, Fmax/BW and total distance run at M6 vs M3 is also reported. All values are expressed as the mean ± SEM from the number of mice indicated in brackets. For BW at M3 or M6, no statistically significant differences were found by ANOVA. Statistically significant differences among groups was found by ANOVA for Fmax (F = 46, p < 1.2 x 10^−11^), Fmax/BW (F = 54, p < 1.2 x 10^−12^) and *e*-test (F = 20, p < 1.4 x 10^−7^). Bonferroni post hoc correction for individual differences between groups is as follows: vs * wt M3 (4 x 10^−8^ < p < 0.001)

^#^ wt M6 (2.8 x 10^−10^ < p < 7.7 x 10^−8^).

**Fig 6:**
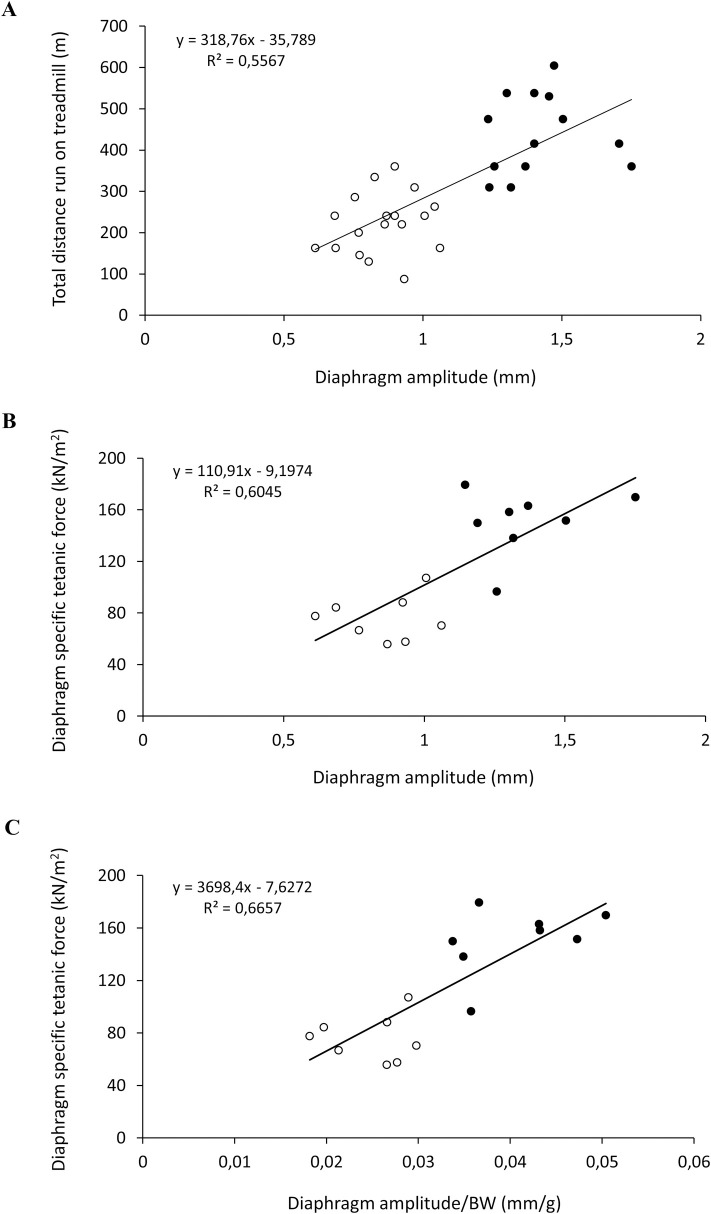
Correlation of diaphragm amplitude with *in vivo* fatigability and *ex vivo* force. In **A** is shown the linear correlation obtained by plotting individual mouse values from wt (●) and *mdx* (○) mice for total distance run (m) during the *e*-test at 3 and 6 months of age, expressed as a function of diaphragm amplitude absolute values (mm) at the same time points. In **B** and **C** is shown the linear correlation obtained by plotting individual mouse values from wt (●) and *mdx* (○) mice at 6 months of age for diaphragm specific isometric tetanic force (sP0, kN/m^2^), expressed as a function of absolute (**B**, mm) or body weight normalized (**C**, mm/g) diaphragm amplitude.

At the same time points, an acute exercise resistance test (*e*-test) was performed to assess *in vivo* fatigue, as an important index of neuromuscular function (**Table 2**). As can be seen, *mdx* mice run significantly less than wt mice both at M3 and M6. In parallel, *mdx* mice at M6 showed a reduction in exercise performance equal to– 32% with respect to M3, while wt mice showed a notable increase of + 22.1% for this parameter. A correlation between total distance run (*e*-test) by wt and *mdx* mice and diaphragm amplitude at M3 and M6 was also calculated, showing a coefficient of correlation R^2^ of 0.56 (**Fig 6A**).

### Isometric and eccentric contractile parameters of isolated diaphragm

**Fig 7:**
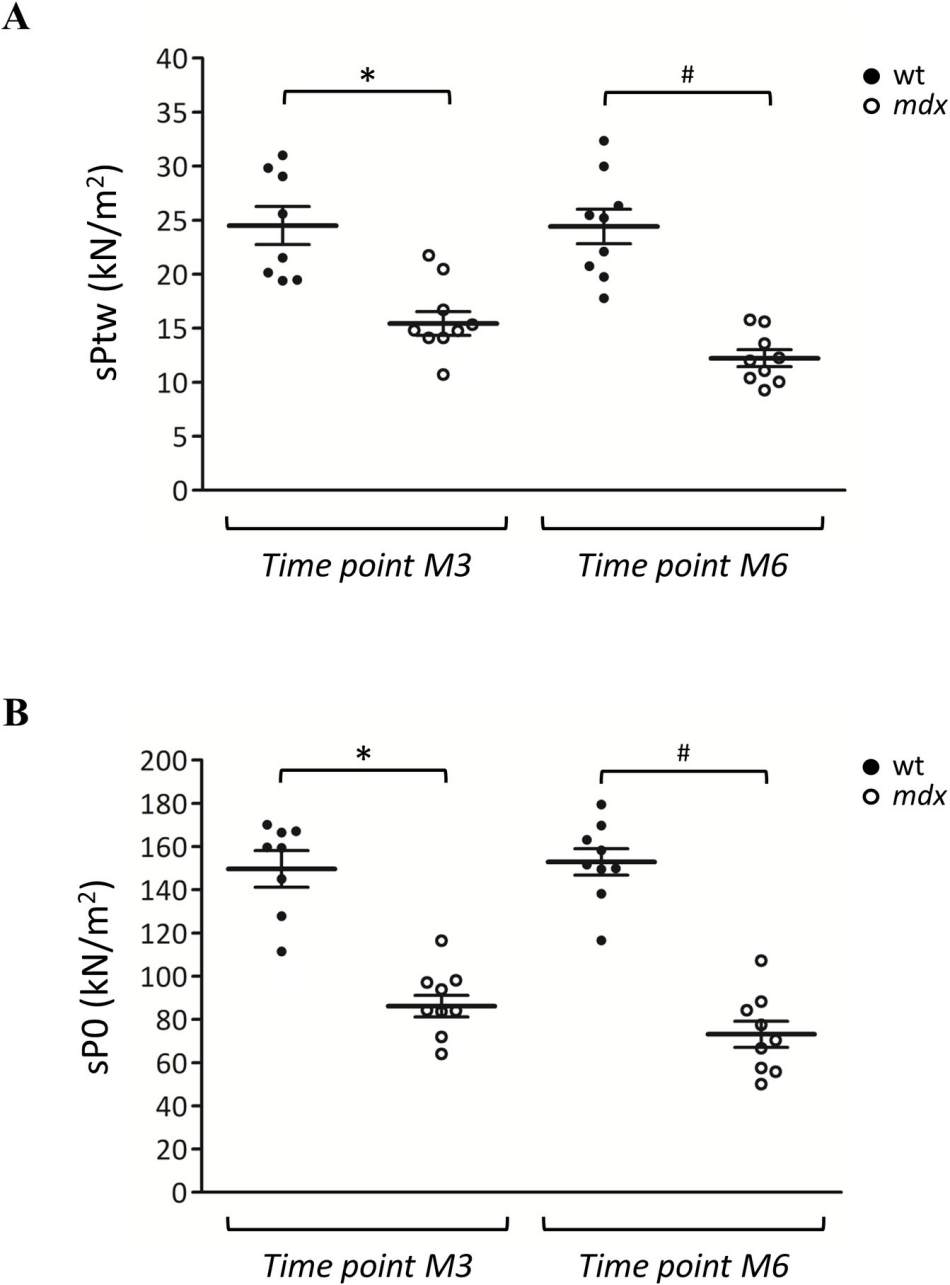
Diaphragm *ex vivo* isometric contraction. The scatter plot graphs in **A** and **B** show individual normalized values, mean, and standard error of the mean (SEM) for maximal isometric twitch (**A**, sPtw measured in kN/m^2^) and tetanic (**B**, sP0 measured in kN/m^2^) tension of diaphragm strips isolated from wt (●) and *mdx* (○) mice at 3 (M3) and 6 (M6) months of age. In detail, values at M3 were obtained from one cohort of wt and one of *mdx* mice (n = 9 animals *per* group, with one wt excluded from data analysis due to experimental issues), dedicated to *ex vivo* assessments at this time point. Values at M6 were obtained from wt and *mdx* mice of the main experimental groups (n = 9 for each genotype). Statistically significant differences among groups were found by ANOVA for sPtw (F > 16.6, p < 1.5 x 10^−6^) and sP0 (F > 27.6, p < 9.3 x 10^−9^). Bonferroni post hoc correction for individual differences between groups is as follows: significantly different vs * wt M3 (9.9 x 10^−6^ < p < 0.001), ^#^ wt M6 (3.5 x 10^−8^ < p < 1.5 x 10^−6^).

At the end of the *in vivo* experimental phase (M6), an *ex vivo* evaluation of isometric and eccentric contractile properties of diaphragm isolated from wt and *mdx* animals was performed (**Fig 7A and 7B**). In parallel, an independent group of 3-month-old *mdx* mice and one of age-matched wt mice were sacrificed to obtain these values at M3. A significant difference between the two genotypes was observed for both diaphragm specific twitch (sPtw, kN/m^2^, **Fig 7A**) and tetanic (sP0, kN/m^2^, **Fig 7B**) force values, in line with our previous results [13, 17, 23–25]. In fact, sPtw and sP0 from dystrophic mice were significantly decreased compared to those found in wt mice, both at M3 (sPtw:– 33.2%; sP0:– 49.9%) and M6 (sPtw:– 40.4%; sP0:– 51.5%) (**Fig 7A and 7B**). Interestingly, we found a good linear correlation (R^2^ = 0.60) between diaphragm movement amplitude, measured by ultrasonography, and diaphragm sP0 from same mice (**Fig 6B**). A similar correlation coefficient (R^2^ = 0.66) was also found between diaphragm amplitude normalized to body weight and diaphragm sP0 (**Fig 6C**).

Since respiratory acts are mainly characterized by stretch-like movements, the isometric force drop of isolated diaphragm after eccentric contractions was also assessed. Results obtained at M6 are reported in **S1A–S1C Fig**. In *mdx* mice, diaphragm force showed a significant percentage of reduction at the 5^th^ eccentric contraction compared to wt (**S1A Fig**). After 4 minutes from the end of the eccentric stimulation protocol, the percentage of force recovered with respect to the value obtained prior to the initiation of the protocol, was significantly lower in *mdx* mice compared to wt, which showed an almost complete recovery of isometric force, as expected [23]. This difference between the genotypes remained statistically significant even after 30 min (**S1B Fig**). However, *mdx* diaphragm was less compliant to stretch with respect to that of wt mice, as shown by significantly lower values obtained by calculating the difference in isometric force produced before and after the stretch for each eccentric stimulus (**S1C Fig**). This observation might be due to an altered elastic capacity of dystrophic diaphragm, likely related to the progressive increase of muscle fibrosis in *mdx* animals [1].

### TGF-β1 levels and histopathology in diaphragm

**Fig 8:**
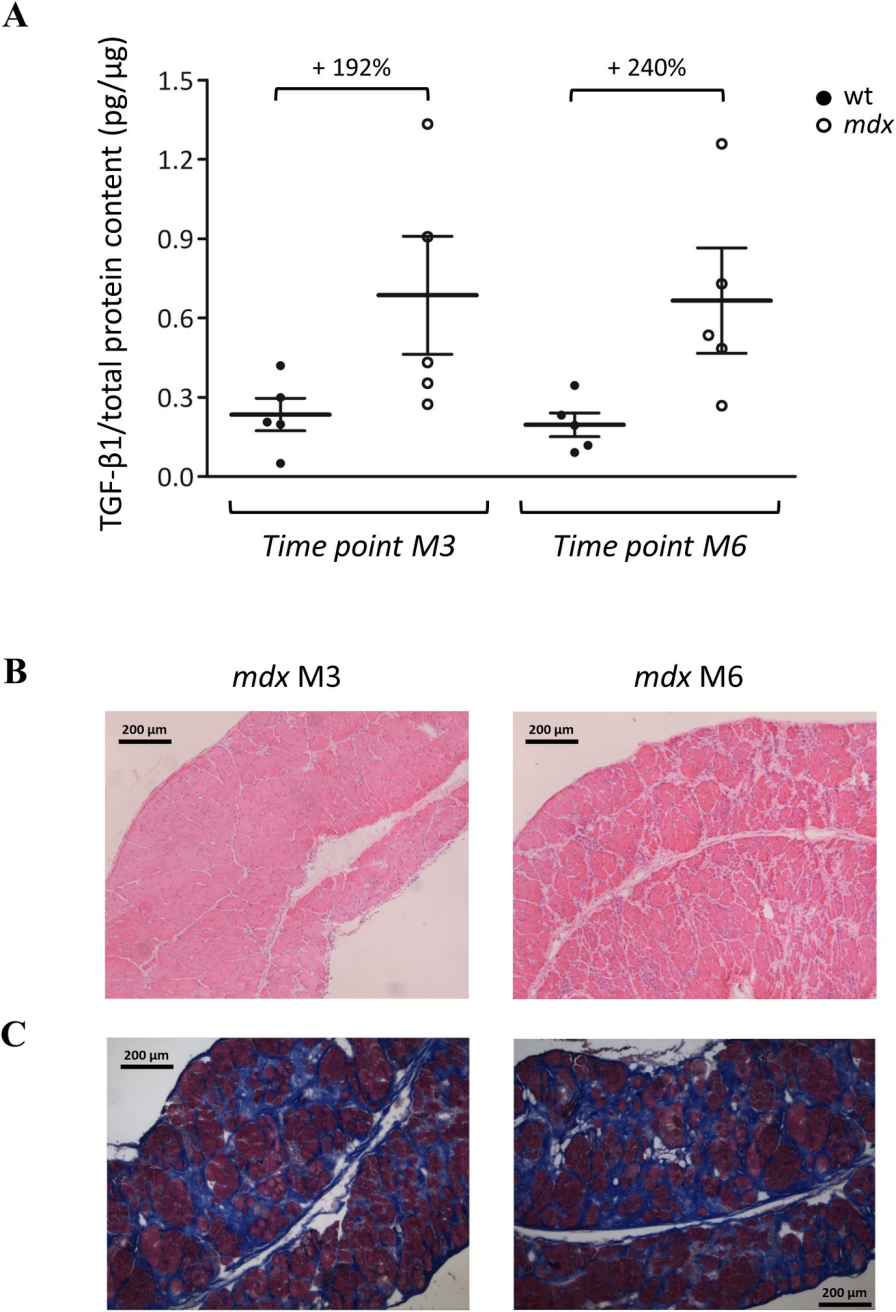
Diaphragm levels of pro-fibrotic marker TGF-β1 and histopathology. The scatter plot graph in **A** illustrates individual mouse values, mean and standard error of the mean (SEM) for TGF-β1 levels normalized to total protein content (pg/μg), measured by ELISA in diaphragm samples from wt (●) and *mdx* (○) mice at 3 (M3) and 6 (M6) months of age. No statistically significant differences among groups were found by ANOVA followed by Bonferroni post hoc correction. The percentage increase of TGF-β1 levels calculated in M3 and M6 *mdx* mice with respect to age-matched wt is indicated above the bars. In **B** are shown representative sections of hematoxylin & eosin stained DIA muscles from *mdx* mice at M3 and M6 (10× magnification). Images are characterized by a disorganized tissue architecture with the presence of centronucleated fibers, inflammatory infiltrates and areas of non-muscle tissue, typical signs of dystrophic histopathology. In **C** are shown representative sections of DIA muscles from dystrophic mice at M3 and M6 (10× magnification) stained with Masson’s trichrome. The staining allows to discriminate nuclei (dark brown), muscle fibers cytoplasm (pink-red), collagen and bone (blue).

In order to reinforce our data on echodensity as an index of fibrosis occurring in *mdx* mice diaphragm, we evaluated by ELISA the expression of the cytokine TGF-β1, a key pro-fibrotic biomarker, in diaphragm samples from M3 and M6 *mdx* mice, in comparison to age-matched wt (**Fig 8A**). In agreement with previously published data [23, 24], in *mdx* animals, total TGF-β1 levels normalized to total protein content were consistently higher than those observed in wt. In detail, M3 *mdx* mice showed an increase in total TGF-β1 concentration of +192% compared to age-matched wt, and this percentage raised to +240% at M6 (**Fig 8A**). The lack of statistical significance is likely related to the high inter-individual variability between samples. Moreover, the fibrotic change was also confirmed by the qualitative analysis of both M3 and M6 *mdx* diaphragm through H&E staining, showing an increase in non-muscle area at M6 time point compared to M3 and to the wt counterpart (**Fig 8B**). Fibrotic areas were further evidenced by the more specific Masson’s trichrome staining performed on both M3 and M6 *mdx* diaphragm sections (**Fig 8C**). The quantitative morphometric analysis of these sections evidenced a percentage of fibrotic areas of 10.6 ± 3.3% in M3 *mdx* mice (n = 4), and of 24.4 ± 3.7% in M6 *mdx* mice (n = 5).

## Discussion

The improvement of preclinical translational research and the identification of novel therapeutics benefit from both continuously growing knowledge on molecular pathology mechanisms and target properties, as well as from methodological advancements. This allows to optimize drug design by enhancing selectivity and specificity, while the use of integrated approaches such as *in silico* methods, 3D *in vitro* tissue models and non-invasive *in vivo* experiments, help to predict benefit and efficacy without an extensive use of laboratory animals [28–32]. In fact, the use of non-invasive *in vivo* techniques in preclinical studies conducted in reliable animal models of human pathological conditions, offers the possibility to perform a longitudinal monitoring of the same animal throughout the experimental window, thus allowing to minimize the inter-individual differences in response to drugs [3, 14, 15]. The need to improve the reliability of preclinical studies is particularly relevant in the field of rare neuromuscular diseases, such as DMD, where the complex pathogenic cascade makes target-addressed drug design still poor [2–4]. Nonetheless, many drugs that seemed promising in *mdx* mice then failed in the clinical setting; this, further pushed the international scientific community working on DMD to put efforts into the identification and optimization of reliable preclinical readouts of diagnostic and therapeutic interest, possibly by means of non-invasive and longitudinal methods of clinical relevance [1–5].

According to these general aims, in the present study we carried out a longitudinal ultrasonography evaluation of diaphragm muscle function and morphology in *mdx* mice at two crucial time points for preclinical studies, 3 and 6 months of age, in order to independently confirm and, possibly, to further increase the interest in an early-use of the ultrasound technique, as a reliable *in vivo* outcome for translational research on DMD. This is of particular importance since ultrasonography offers several advantages, such as low costs and the possibility to be carried out at the bedside of the patient; moreover, it is particularly useful in cases of children and/or patients with mobility limitations, conditions often associated to DMD.

To date, the main cause of death in DMD boys is still represented by failure of cardiorespiratory function [33]. Importantly, similarly to patients, *mdx* mice develop a significant cardiomyopathy in a later stage of the disease, between 9 and 12 months of age [11]. Also, *mdx* diaphragm is characterized by a progressive structural and functional alteration, which onsets earlier and with a greater severity than in hind limb muscles, more closely resembling affected subjects’ condition [1]. Precisely, in the *mdx* mouse, the first signs of diaphragmatic degeneration occur around the 30^th^ day of life. At 6 months of age, *mdx* diaphragm is characterized by significant morphological alterations including a reduction in fiber size, necrosis, increased connective tissue and fibrosis [34–38]. The progression of diaphragm impairment in *mdx* mice can be detected *in vitro* on isolated strips, while non-invasive plethysmography in non-anesthetized *mdx* animals is a useful approach to assess the impairment in respiratory function *in vivo*. However, controversial results are available for this technique, likely in relation to the multiple systems involved in the complex control of respiratory function [38, 39]. Consequently, other non-invasive *in vivo* methods, such as ultrasonography, magnetic resonance imaging and spectroscopy, which allow combined and longitudinal assessments of both structural and functional changes in the same *mdx* animals, attract increasing interests and deserved effort of standardization [7–10, 14, 40, 41].

Diaphragm ultrasound is a promising tool for helping to directly assess diaphragm dysfunction in *mdx* mice. In their study, Whitehead *et al*. described a reduction of *mdx* diaphragm movement amplitude, which was well-correlated with specific diaphragm force measured in the same mice *ex vivo* [5]. According to Whitehead’s study, our experiments showed a significant reduction of diaphragm amplitude in 3-month-old *mdx* mice compared to age-matched wt mice. Moreover, the decrease in diaphragm amplitude found in 6-month-old *mdx* mice in comparison to age-matched wt, was similar to that observed at 3 months of age. Interestingly, we found a good correlation between diaphragm movement amplitude and specific tetanic force of isolated diaphragm (sP0). This was maintained when sP0 was correlated with diaphragm amplitude normalized to body weight, indicating that the specific animal size does not influence these parameters. In parallel, diaphragm strips isolated from 6-month-old *mdx* mice were less compliant to stretch than those from wt counterparts, as shown by data on dynamic stiffness and recovery from eccentric contraction, in agreement with previous evidences collected on EDL muscle [13]. Then, the significant reduction in diaphragm amplitude observed in 3-month-old *mdx* mice is in line, and can be explained by, the early degeneration of this respiratory muscle. In parallel, specific alterations in diaphragm architecture, such as fiber branching and morphological heterogeneity could also play a key role in defective force generation at this age [37]. In light of these observations, our data validated previous results by Whitehead’s group, further supporting the measurement of ultrasound diaphragm amplitude as an useful readout to be used in preclinical setting for the assessment of dystrophic pathology progression in *mdx* mice. Furthermore, we also found a good correlation between diaphragm amplitude and *e-test* values obtained *in vivo* at M3 and M6, indicating that the impaired running performance may be, at least in part, attributable to the progressively impaired respiratory function in addition to the main involvement of an increased fatigability of hind limb muscles. In fact, according to previous studies, no functional or structural alterations were detected at cardiac level by ultrasonography in *mdx* animals at both M3 and M6 [11, 17].

Importantly, besides this independent validation of the technique, which is of great relevance in the view of optimizing standardization and reproducibility of methods used, our study presented a major novelty. For the first time, we indeed carried out an assessment of diaphragm muscle echodensity in wt and *mdx* mice, with the aim to introduce a novel and highly valuable marker of disease progression in translational research, as it is easily translatable into clinical settings. In fact, an increment in echodensity is most likely related to an increased amount of non-muscle tissue, *i*.*e*. adipose tissue and fibrosis. Our experiments evidenced an increment, albeit not significant, in diaphragm echodensity in 3-month-old *mdx* compared to wt mice of the same age. In 6-month-old *mdx* mice we found a significantly increase of diaphragm echodensity with respect to age-matched wt; this latter result is in line with the age-dependent increase in collagen content in *mdx* diaphragm described elsewhere, which in turn parallels that observed in patients. This, however, seems to play a complex role in muscle stiffness with possible minimal effect on passive one [42, 43].

Our first-time observations in the *mdx* mouse model are in agreement with other promising echodensity data recently obtained by ultrasound experiments in the Golden Retriever Muscular Dystrophy canine model (GRMD), where the increase of this index appeared to be strongly correlated to the percentage of interstitial fibrous tissue in muscle biopsies [44]. In parallel, ultrasound echodensity measurements performed on the diaphragm of a canine model of X-Linked Myotubular Myopathy, resulted as a valid approach to determine diaphragm function in patients affected by neuromuscular disorders [45]. In addition, the recent evaluation of ultrasound echodensity in skeletal muscles of young DMD patients, showed to be well-correlated with clinical parameters commonly associated with disease progression (*i*.*e*. functional grading scores, muscle strength, ambulatory status and motor ability), further supporting our hypothesis that this method, when performed by trained examiners, can be effective in monitoring the evolution of symptomatology in affected subject [7–10].

Overall, our ultrasonography results indicate that the measurement of echodensity is capable to detect an age-dependent variation of this parameter in *mdx* mice. Importantly, our analysis was also aimed to evaluate a possible attenuation of ultrasonography signal by the abdominal wall. The very weak negative correlation found by the attenuation analysis, sustains our hypothesis that the observed changes in echogenicity are solely related to an alteration in diaphragm morphology, although the method does not allow to get information on the type of the hyperechoic tissue specifically accounting for these changes.

Then, the significant reduction of diaphragm movement amplitude in the presence of a minor increase of echodensity observed in 3-month-old *mdx* mice, can be associated to the well-known complex pathological cascade occurring in both limb muscles and diaphragm in the early disease phase, such as inflammation, oxidative stress, activated calcium-dependent proteolytic events and progressive mechanical-metabolic maladaptation [23, 24, 32, 36, 46–50]. By contrast, later changes in diaphragm function and echogenicity are more directly linked to progression of fibrosis. In fact, the quantification of fibrotic tissue performed by histological analysis was in line with the echodensity results, confirming the clear age-dependent trend of increase in *mdx* diaphragm. This was also supported, although indirectly, by the consistent age-dependent increase in the levels of the pro-fibrotic cytokine TGF-β1.

## Conclusions

Our study corroborates the usefulness of ultrasound imaging as a tool to monitor *mdx* mice dystrophic pathology progression since early stages, with minimal invasiveness. In particular, our first-time observations on diaphragm echodensity, together with the independent validation of diaphragm movement amplitude and morphometric evaluation in the *mdx* mouse, represents the strength of our study. In fact, this provides novel evidence about the usefulness of the method to perform a longitudinal evaluation of dystrophic diaphragm pathology progression, and particularly of fibrosis, and possibly of the efficacy of therapeutic interventions in preventing or slowing this process in this or other murine models of DMD. This is of importance for both clinically-oriented preclinical research and for trials on DMD patients.

The complexity to find direct evidence of correlation in some of the parameters analyzed herein, could be due to both different methodologies used and to the typical variability observed in *mdx* mice. However, the good correlation found with diaphragm *ex vivo* functional parameters and the lack of a parallel cardiac involvement at this age, clearly demonstrate the possibility to use this technique as a valid experimental method to assess purely diaphragm dysfunction in dystrophic mice.

## Supporting information

S1 File (DOCX)

S1 Fig Diaphragm response to eccentric contraction and elastic properties. The histograms in **A**, **B** and **C** show the modifications of *ex vivo* isometric force in response to eccentric contractions in isolated diaphragm strips from wt and *mdx* mice at 6 months of age (M6). Each bar is the mean ± SEM from 8 animals *per* group (with one wt and one *mdx* excluded from data analysis due to experimental issues). In **A** is shown the percentage reduction of diaphragm isometric tension, calculated at the 5^th^ eccentric contraction. In **B** is shown the percentage recovery of diaphragm isometric force measured after 4 and 30 minutes (min) from the eccentric contraction protocol. In **C** is shown the calculation of diaphragm stiffness (mN/mm^3^) at each eccentric stimulus. *A significant difference between genotypes was found by unpaired Student's t-test (0.0001 < p < 0.03).
(TIF)
