# Supplementary material for: Ultrasonography validation for early alteration of diaphragm echodensity and function in the mdx mouse model of Duchenne muscular dystrophy
Source: PLoS One. 2021 Jan 12;16(1):e0245397. doi: 10.1371/journal.pone.0245397 (PMC7802948; doi:10.1371/journal.pone.0245397)
Supplement: S1 File — (DOCX) [file pone.0245397.s001.docx]

**Link to download the code, as HTML file of the Jupyter Notebook, used to verify ultrasound attenuation from abdominal wall thickness.**

<https://drive.google.com/file/d/1QHt4mF3yhf119ryfhfLWw35vCxZA4Wy4/view?usp=sharing>
