# Supplementary figures and images for: Ultrasonography validation for early alteration of diaphragm echodensity and function in the mdx mouse model of Duchenne muscular dystrophy
Source: PLoS One. 2021 Jan 12;16(1):e0245397. doi: 10.1371/journal.pone.0245397 (PMC7802948; doi:10.1371/journal.pone.0245397)

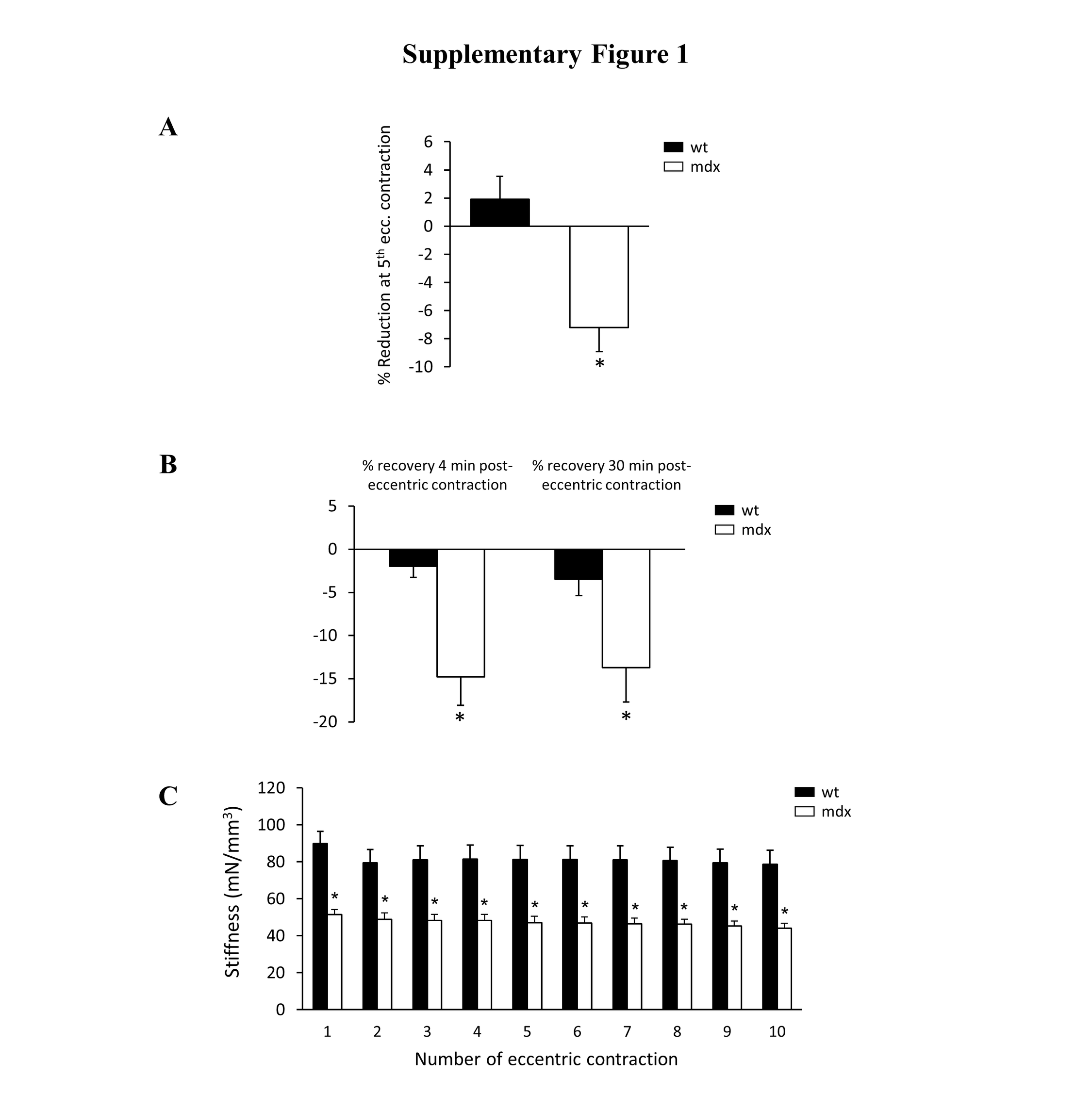

Supplement: S1 Fig — The histograms in A, B and C show the modifications of ex vivo isometric force in response to eccentric contractions in isolated diaphragm strips from wt and mdx mice at 6 months of age (M6). Each bar is the mean ± SEM from 8 animals per group (with one wt and one mdx excluded from data analysis due to experimental issues). In A is shown the percentage reduction of diaphragm isometric tension, calculated at the 5th eccentric contraction. In B is shown the percentage recovery of diaphragm isometric force measured after 4 and 30 minutes (min) from the eccentric contraction protocol. In C is shown the calculation of diaphragm stiffness (mN/mm3) at each eccentric stimulus. *A significant difference between genotypes was found by unpaired Student's t-test (0.0001 < p < 0.03). (TIF) [file pone.0245397.s002.tif]
